# From populations to molecules: a life in food and health

**DOI:** 10.1038/s41430-021-01002-4

**Published:** 2021-10-21

**Authors:** Michael J. Gibney

**Affiliations:** Institute of Food and Health, University College Dublin, Dublin, Ireland

**Keywords:** Nutrition, Biomarkers



## Getting started

When I was a young teenager in the 1960s, Ireland was embarking on a new industrialised pathway where the food industry was central. I wanted to study food science but no such degrees existed in Dublin at the time and so I opted to study Agricultural Science, specialising in Chemistry. In hindsight, this was an odd speciality to pick since, for my final exams in secondary school I studied Irish, English, French, Latin, physics and mathematics. I had never studied either biology or chemistry both of which would be central to my university studies but, it turned out that not only did I really enjoy these new subjects, but I was good at them. I had a wonderful 5 years at University College Dublin where I was active in left wing politics and was a keen debater. Based on my final exam marks I was awarded a scholarship which I used to complete a master’s degree working on the dietary determinants of branched chain fatty acids in the adipose tissue of lambs. I enjoyed research and decided to pursue a PhD. I applied to many universities and was offered places at Cambridge, Wageningen, Calgary and Sydney. At that time, Sydney University Veterinary School was one of the global leaders in several areas of animal science: nutrition, genetics and reproduction. I accepted an offer of a PhD with the academic duties of a Teaching Fellow.

In January 1973, my wife and I arrived in Sydney and I began my PhD working on the digestive physiology of neonatal lambs. The lambs had dual canulae fitted at the jejunum and distal ileum with the digestive flow exteriorised through a vinyl canula fitted with a sampling tap. The lambs were fed on experimental milks. It was hard work, 7 days a week during the long season from spring to autumn. Both my MSc and PhD training, gave me a passion about experimental design, analytical chemistry and biology and statistics all of which would serve me well as a foundation for my future career. In 1976, my wife Jo, pregnant on our third child, and I returned to Ireland where I took up a post-doctoral post in the Agricultural Institute in Dublin. It was not a particularly stimulating post and neither myself or Jo were especially happy. I began to dabble in human nutrition using household budget and consumer price data and, I began to look around for something more challenging.

## From animal to human nutrition

I applied for and was offered a post as a lecturer in human nutrition at the University of Southampton Medical School which I took up in 1997. In Sydney, I had lectured students of agriculture and veterinary medicine but also, students of human nutrition and dietetics and that experience was key to making the switch from animal to human nutrition. Whilst working with neonatal lambs at Sydney, we were tempted to follow up on UK research suggesting that calves fed on milk substitutes based on isolated soy protein, developed systemic IgG antibodies to this protein. Now, in a medical school with state-of-the-art facilities and support, I began to study, in both rabbit models and in humans, the effect of dietary proteins on both secretory and systemic immune responses [1]. I also returned to the main area of my MSc studies, lipid metabolism [2].

After 8 wonderful years at Southampton, an opportunity came up to return to Ireland and I successfully applied for an academic post at the Medical School of Trinity College Dublin, based at St James hospital. As part of the Department of Clinical Medicine, my research would now embrace the interface of nutrition with cardiology, gerontology, paediatrics, haematology and gastroenterology and of course my own personal interest in postprandial lipid metabolism [3–8]. I was tasked with establishing a new BSc degree in human nutrition and dietetics and I would spend the next quarter of a century at Trinity.

## The European Union dimension

Shortly after taking up my post, I was asked to join a European Union (EU) scientific consortium, named Euronut. Professor Jo Hautvast from Wageningen University in the Netherlands, also a contributor to this crystal ball series, was chair and he became a role model for me [9]. As far as I could tell, Jo rated his success on how he fostered others to achieve their goals and I tried to emulate that throughout my career. Jo was the most visionary person I ever worked with. Not long afterwards, I was appointed to the Scientific Committee for Food (SCF), the highest level scientific advisory committee to the EU, covering the toxicology and nutritional issues of food. I would spend 15 years on the SCF and when all the advisory committees were combined into a single Public Health Steering Committee, I was one of the 12 members chosen to serve on this new entity. Because of my prior experience in managing committees in the complex food regulatory environment and because I had no prior interest in the biology of prions, I was asked to chair the working group on BSE, during the height of that public health crisis. Chairing that working group, with the world’s top experts in the biology of prions was challenging but I managed to conclude the work without losing any friends. It was a remarkable experience and I was joined on that committee by another contributor to this crystal ball series, Professor Philip James [10].

All of this involvement with EU colleagues led to my becoming involved in competitive EU research funds and over a period of 20 years, I was involved in eight such consortia and was the principal investigator in five. The last of those was Food4Me, a 14 million-euro project on personalised nutrition. The story of how I got involved in this field of molecular nutrition is worth relating. I was a member of a high level advisory committee to the Nutrition Research Centre of Nestle in Lausanne and in 1999, myself, Vernon Young from MIT and Bruce German from Davis were asked to put together a series of seminars on whatever hot topics in nutrition we thought would shape future research in this field. We invited the two top experts in the metabolomics of pharmacology and toxicology, Jeremy Nicholson from Imperial College and Jan van der Greef from the University of Leiden. That evening we dined in a Chinese restaurant and I tried to get my head around the bewildering theory of partial least-squares discriminant analysis (PLS-DA), otherwise known as pattern recognition. Jan van der Greef was a wonderful and patient tutor and took me through all elements of PLS-DA. When the meal was over, I took home the paper tablecloth on which many scribbles and diagrams had been added. I knew then that this science of metabolomics, so advanced in pharmacology and toxicology, would have a huge role to play in molecular nutrition [11]. The Food4Me study and the European Nutrigenomic Organisation (NuGo), organised by Ben van Ommen from TNO in Leiden, would give Europe a big lead in the field of molecular nutrition.

Besides research I involved myself in the development of nutritional science at the collegiate level. I served as President of the Nutrition Society, headquartered in London, from 1995 to 1998. As someone who very much enjoyed teaching, I was frustrated by the lack of comprehensive textbooks in the field, each one serving some aspects of a curriculum but none covering all the needs of a student of human nutrition. When I completed my term as President, I took on the role of founding editor of a suite of textbooks on nutrition which were published as the Nutrition Society Textbook Series and these are now used in universities across the world.

## The twilight zone

Now, in my 8th year of retirement, I have time to reflect on the field of nutrition and, frankly, I am concerned. We have made little progress in mastering accurate measures of food intake, something absolutely fundamental to the subject. Energy underreporting, which is found in about 40% of respondents in any typical survey, is a major source of error in public health nutrition. Being female, overweight and socially disadvantaged are among the main drivers of energy underreporting. Epidemiologists manage this phenomenon by applying various statistical adjustments in multi-regression analysis such as body mass index, gender, social class etc. But the problem is that these adjustments are based on theory. I know of no large-scale study that quantified energy underreporting using for example, doubly labelled water and also conducted an open search of factors that might influence underreporting. Epidemiologists must also adjust for other confounding factors that might influence the end point in question. For example, if the measured outcome is cardiovascular disease, then it makes sense to control for any family history of heart disease. But our eating habits are shaped by many things. Bereavement, divorce and unemployment all influence dietary habits as does social isolation and loneliness [12–15]. These, among many other possibilities, ought to be considered in nutritional epidemiology.

A second major failure of nutrition is the manner in which we record and report food intake. Any large national dietary survey that uses self-reported food choice as opposed to the fixed choice of food frequency approaches, will tell us the mean daily intake of upwards of 3000 individual foods. But if you wanted to know the mean daily intake of ham sandwiches, you would be disappointed. The argument is made that whereas we can compute the mean daily intake of white bread, brown bread, butter, margarine, mayonnaise and ham, we can’t tell how they were combined to form a ham sandwich. That is because we deconstruct reported meals into their individual food components since it is at the food level that nutritional composition data are available. White bread, brown bread, butter, margarine, mayonnaise, mustard and ham are the foods we combine to make ham sandwiches and with these foods, there are 24 possible variations of ham sandwiches. And if someone suggests that lettuce might be added to the mix, we are up to 48 varieties and if cheese is thrown in, we reach 96 variations. Quite simply, it is impossible to assign a nutritional composition to every possible variety of a ham sandwich. But if the time of an eating occasion is recorded, we can package all the food codes of that eating occasion into brackets. Now we have a bracketed digital code for a given eating occasion and when all eating occasions are considered we might have about six sets of bracketed digital codes to cover a single day. It cannot be beyond the competence of data analysts to seek patterns in data. Myself and my then post doc Áine Hearty attempted this some years ago with little success [16]. But then, we are not in the informatics business. People eat meals and if we are to modify food intake we must understand how foods are combined into meals and meals into dietary patterns. Take for example a study which shows an association between consumption of say, avocados and body mass index. The researchers have no notion of the meal context in which avocados were consumed and no way of knowing the extent to which avocado intake is positively or negatively associated with other foods. Avocado intake may simply be a marker for particular eating patterns. Developing meal patterns is challenging but it is a goal so worthy of scholarly pursuit [17].

A final look into my crystal ball would be to nutrition what CERN is to particle physics. There will come a day when meal pattern analysis is fully operational and we can explore national food consumption data for meal patterns. Let us imagine that we can say with confidence that 95% of the variation in food choice can be explained by say 10 meal patterns. Volunteers could be rotated through each of these 10 meal patterns in a randomised crossover design with blood, urine and faecal samples collected for metabolomic analysis. The subjects would be housed in purpose built metabolic wards with all their food and beverages strictly controlled. Each dietary pattern would create a defined metabolic fingerprint, all of which would be entered into a publicly available database. When you visit your doctor, you will bring along a urine and faecal sample. These along with your blood would be shipped off to a metabolomics or proteomics facility, your fingerprint would be entered into the database and your doctor would be able to then tell you which dietary pattern you belong to. On that basis, you would get meal-based dietary advice to optimise your diet [18]. Whenever I raise this with colleagues I am told it will never happen. Somehow, I think that the same negative response was given to the French physicist Louis de Broglie when, in 1949, he first proposed the development of CERN. If nutritional science does not conceptualise, debate, discuss and promote the concept of very large, complex, ambitious and expensive studies nutrition studies, then they will never happen.

I have had a wonderful and fulfilling career in nutrition which, approaching my mid- seventies, is still operational. Of all the people who helped and encouraged me, who patiently listened to seemingly daft ideas and who shepherded me to safety when I was veering down a wrong path, the stand out person is my wife Jo. In the late 1980’s when I began to be responsible for large EU collaborative projects, I needed an organiser and Jo left her safe job in a bank to take on the risk of managing the many meetings and the financial accounts of these pan-EU projects. We worked together for 27 years and as I write we are in our 50th year of marriage. Thanks Jo.

## References

[1] Gibney MJ, Gallagher PJ, Sharratt GP, Benning HS, Taylor TG, Pitts JM (1980). Antibodies to heated milk protein in coronary heart disease. Atherosclerosis..

[2] Gibney MJ, Bolton-Smith C (1988). The effect of a dietary supplement of n-3 polyunsaturated fat on platelet lipid composition, platelet function and platelet plasma membrane fluidity in healthy volunteers. Br J Nutr.

[3] Cooke T, Sheahan R, Foley D, Reilly M, D’Arcy G, Jauch W (1994). Lipoprotein(a) in restenosis after percutaneous transluminal coronary angioplasty and coronary artery disease. Circulation..

[4] Tully AM, Roche HM, Doyle R, Fallon C, Bruce I, Lawlor B (2003). Low serum cholesteryl ester-docosahexaenoic acid levels in Alzheimer’s disease: a case-control study. Br J Nutr.

[5] Freeman VE, Mulder J, van’t Hof MA, Hoey HM, Gibney MJ (1998). A longitudinal study of iron status in children at 12, 24 and 36 months. Public Health Nutr.

[6] Dowling S, Mulcahy F, Gibney MJ (1990). Nutrition in the management of HIV antibody positive patients: a longitudinal study of dietetic out-patient advice. Eur J Clin Nutr.

[7] Kearney J, Kennedy NP, Keeling PW, Keating JJ, Grubb L, Kennedy M (1989). Dietary intakes and adipose tissue levels of linoleic acid in peptic ulcer disease. Br J Nutr.

[8] Shishehbor F, Roche HM, Gibney MJ (1998). The effect of acute carbohydrate load on the monophasic or biphasic nature of the postprandial lipaemic response to acute fat ingestion in human subjects. Br J Nutr.

[9] Hautvast J (2017). A professional life in nutrition sciences and training. Eur J Clin Nutr.

[10] James W (2017). A clinical nutritionist’s experience and expectations. Eur J Clin Nutr.

[11] Gibney MJ, Walsh M, Brennan L, Roche HM, German B, van Ommen B (2005). Metabolomics in human nutrition: opportunities and challenges. Am J Clin Nutr.

[12] Rosenbloom CA, Whittington FJ (1993). The Effects of Bereavement on Eating Behaviors and Nutrient Intakes in Elderly Widowed Persons. J Gerontol.

[13] Yannakoulia M, Papanikolaou K, Hatzopoulou I, Efstathiou E, Papoutsakis C, Dedoussis GV (2008). Association Between Family Divorce and Children’s BMI and Meal Patterns: The GENDAI Study. Obesity.

[14] Dave DM, Kelly IR (2012). How does the business cycle affect eating habits?. Soc Sci Med.

[15] Walker D, Beauchene RE (1991). The relationship of loneliness, social isolation, and physical health to dietary adequacy of independently living elderly. J Am Diet Assoc.

[16] Hearty AP, Gibney MJ (2008). Analysis of meal patterns with the use of supervised data mining techniques-artificial neural networks and decision trees. Am J Clin Nutr.

[17] O’Hara, C and Gibney, ER Meal Pattern Analysis in Nutritional Science: Recent Methods and Findings, Advances in Nutrition, 2021, nmaa175, 10.1093/advances/nmaa175. 10.1093/advances/nmaa175 PMC8321870 33460431

[18] Gibney M, Allison D, Bier D, Dwyer J (2020). Uncertainty in human nutrition research. Nat Food.

